# High levels of mortality, malnutrition, and measles, among recently-displaced Somali refugees in Dagahaley camp, Dadaab refugee camp complex, Kenya, 2011

**DOI:** 10.1186/1752-1505-7-1

**Published:** 2013-01-22

**Authors:** Jonathan A Polonsky, Axelle Ronsse, Iza Ciglenecki, Monica Rull, Klaudia Porten

**Affiliations:** 1 Epicentre, 53-55 rue Crozatier, Paris, France; 2 Médecins Sans Frontières Operational Centre Geneva, 78 rue de Lausanne, 1211, Genève 21 CP 116, Switzerland

**Keywords:** Mortality, Malnutrition, Refugees, Measles, Epidemics, Vaccination, Policy

## Abstract

**Background:**

Following a rapid influx of over 200,000 displaced Somalis into the Dadaab refugee camp complex in Kenya, Médecins Sans Frontières conducted a mortality and nutrition survey of the population living in Bulo Bacte, a self-settled area surrounding Dagahaley camp (part of this complex).

**Methods:**

The survey was conducted between 31^st^ July and 10^th^ August 2011. We exhaustively interviewed representatives from all households in Bulo Bacte, collecting information on deaths, births, and population movements during the recall period (15^th^ February 2011 to survey date), in order to provide estimates of retrospective death rates. We recorded the mid-upper arm circumference and presence or absence of bipedal oedema of all children of height 67-<110 cm to provide estimates of global and severe acute malnutrition.

**Results:**

The surveyed population included 26,583 individuals, of whom 6,488 (24.4%) were children aged under 5 years. There were 360 deaths reported during the 177 days of the recall period, of which 186 (52%) were among children aged under 5 years. The crude death rate for the entire recall period was 0.8 per 10,000 person-days. The under-5 death rate was 1.8 per 10,000 person-days. More than two-thirds of all deaths were reported to have been associated with diarrhoea (25%), cough or other breathing difficulties (24%), or with fever (19%). Measles accounted for a reported 17% of all deaths; this was due to a measles outbreak that occurred between June and October 2011.

Global acute malnutrition was observed in 13.4%, and severe acute malnutrition in 3.0%, of children measuring 67-<110 cm. Among children measuring 110-< 140 cm, 9.8% met the admission criteria for entry into the nutritional programme. Trends of decreasing death rates and malnutrition prevalence with length of stay in Bulo Bacte were observed.

**Conclusions:**

We report high death rates and prevalence of malnutrition among this population, reflecting at least a partial failure of the various humanitarian and governmental actors to adequately safeguard the welfare of this population. An outbreak of measles and long delays before registration should not have occurred. The recommendations for measles vaccination among crisis-affected populations should be revised to take into account the epidemiologic context. Organisations must be sensitive and reactive to changes in the health status of the populations they assist.

## Background

Dadaab refugee camp is located in the North Eastern province of Kenya approximately 100 km from the border with Somalia. It was established in 1991, and, with an estimated 472,420 residents as of 8^th^ July 2012 [1], is reported to be the world’s largest refugee camp, comprising three camps: Hagadera, Ifo and Dagahaley.

As a result of the deteriorating humanitarian situation caused by the continued conflict in Somalia and by the failure of rains from October to November 2010, the number of Somalis seeking refuge in Kenya (and elsewhere in the region) has steadily increased. Between January and November 2011, 154,450 individuals were newly-registered in Dadaab, in addition to the estimated 63,000 refugees registered during 2010 [2].

In 2007, the Kenyan government closed the transit and registration facilities in the border town of Liboi due to security concerns, and, starting in August 2008, new arrivals were no longer allocated new plots of land. Without sufficient registration facilities to rapidly process them and without ready housing, the majority of new arrivals settled in the plains surrounding the main camps.

Médecins Sans Frontières has been working in Dagahaley since 2008, providing medical care and psychological assistance to the population living within the camp and its environs, estimated at approximately 123,833 people as of 8^th^ July 2012 [1]. In August 2011, when the survey described herein was conducted, activities included nutritional interventions (in- and out-patient therapeutic feeding centres and supplementary feeding programmes). Médecins Sans Frontières operated programmes in five health posts, six ambulatory and six supplementary centres for nutrition programmes, and a 120-bed second level hospital with a further 200 beds serving as the nutritional stabilisation centre in the hospital. In addition, a network of community health workers actively sought malnourished children and other medical emergencies for referral for treatment.

In order to understand the health status and needs of the then-newly arrived population, Epicentre and Médecins Sans Frontières conducted an exhaustive survey of all households in Bulo Bacte (BB), an area of ‘self-settlement’ outside the camp of Dagahaley, and analysed the data collected during a measles outbreak that affected this population at the time of the survey. In this article, we present the survey estimates of death rates and malnutrition prevalence, and the age and sex breakdown of the suspected measles cases, and discuss the implications of the findings.

## Methods

We conducted an exhaustive household survey in BB between 31 July and 10 August 2011. Enumerators were trained over the course of the three days prior to the survey commencing, with the questionnaire field-tested on the afternoon of the third day. The questionnaire was translated into Somali from the English original, and then back-translated into English to check for consistency in meaning.

All inhabited structures were visited by the survey teams and, when possible, a suitable respondent was identified, with whom the interviews were conducted. Owing to the small size of shelters in this context, the household definition used was “all individuals who regularly eat from the same cooking pot”. Respondents were members of the household aged at least 18 years, and were usually the person identified as the head of the household. The purpose of the survey was explained to all respondents at the outset, and written consent (either signature or thumbprint) recorded for all participating respondents. In the case of there being no potential respondent present at the time of visit, enumerators were instructed to return to the household at the end of that day’s session. If the household was still without potential respondent, the sheet was marked ‘absent’ and left unfilled. The enumerators recorded both the number of ‘absent’ households and the number of households refusing to participate in the survey.

We collected information relating to mortality during the recall period in all eligible households, which was used to calculate retrospective crude and under-5 year death rates. For any death reported during the recall period, information was collected from the household respondent regarding suspected cause or symptoms associated with the death. We also attempted to identify the timing of the death with the aid of a ‘calendar of local events’, which was constructed to help locate the death within the months of the Gregorian calendar. The recall period ran from 15^th^ February 2011 (corresponding to Mawlud, a Muslim festival celebrating the birth of the Prophet Mohammed) to the survey date.

Mid-upper arm circumference measurements were taken, and the presence or absence of bipedal oedema recorded, for all children of height 67-< 140 cm (proxy for children aged 6 months - 9 years [3]) living in included households on the survey date. These data were used to calculate:

a) the age-specific prevalence of severe (SAM) and global (GAM) acute malnutrition for children of height 67-< 87 cm (proxy for children aged 6 – 23 months) and children of height 87-< 110 cm (proxy for children aged 2 – 4 years); and

b) the proportion of children of height 110-< 140 cm (proxy for children aged 2 – 4 years) meeting the admission criteria for entry into the nutritional programme (mid-upper arm circumference <140 mm).

Cochran-Armitage tests-for-trends were performed to explore the effect of duration of stay in BB on prevalence of acute malnutrition [4].

In addition to the above, we also collected information on arrival dates of individuals in BB, and information relating to basic needs that were used to guide the MSF programme response, but which we do not present here.

The study population included all people living in BB. An exhaustive method was selected because we wanted to stratify the results by period of arrival, location, and age-group, while retaining high precision in the results. In addition, this would have, in theory, permitted the detection and referral for treatment of all malnourished children in BB.

The MSF-supported health facilities in Dagahaley compiled a line list, which included information on age, sex, and within-Dagahaley origin for all patients with suspected measles. We analysed this information to describe the outbreak among residents of BB in terms of sex and age.

Data were double-entered into EpiData 3.1 software (The EpiData Association, Odense, Denmark) by two teams of two data entry clerks, and were subsequently cleaned and analysed using StataSE 11.0 (Stata Corporation, College Station, Texas, USA).

Death rates are expressed as deaths per 10,000 person-days, and calculated using mid-point population estimates for the denominator, taking into account deaths, births, departures and arrivals [5]. We calculated the prevalence of acute malnutrition using the definition that any eligible child with mid-upper arm circumference below 115 mm and/or bipedal oedema is severely acutely malnourished, while any eligible child with mid-upper arm circumference 115-< 125 mm without bipedal oedema is moderately acutely malnourished [6,7].

Specific ethical approval for this survey was not sought, as the primary reason for conducting it was to guide MSF operations targeting the newly-arrived refugees in BB.

## Results

Between 31 July and 10 August 2011, 5,119 households were visited, of which 46 (0.9%) did not have a potential respondent present at the time of visit, and were therefore excluded from inclusion in the survey. The surveyed population included 26,583 individuals, of whom 6,488 (24.4%) were children aged under 5 years. The mean household size was 5.3 members. No households refused to participate in the study.

**Figure 1:**
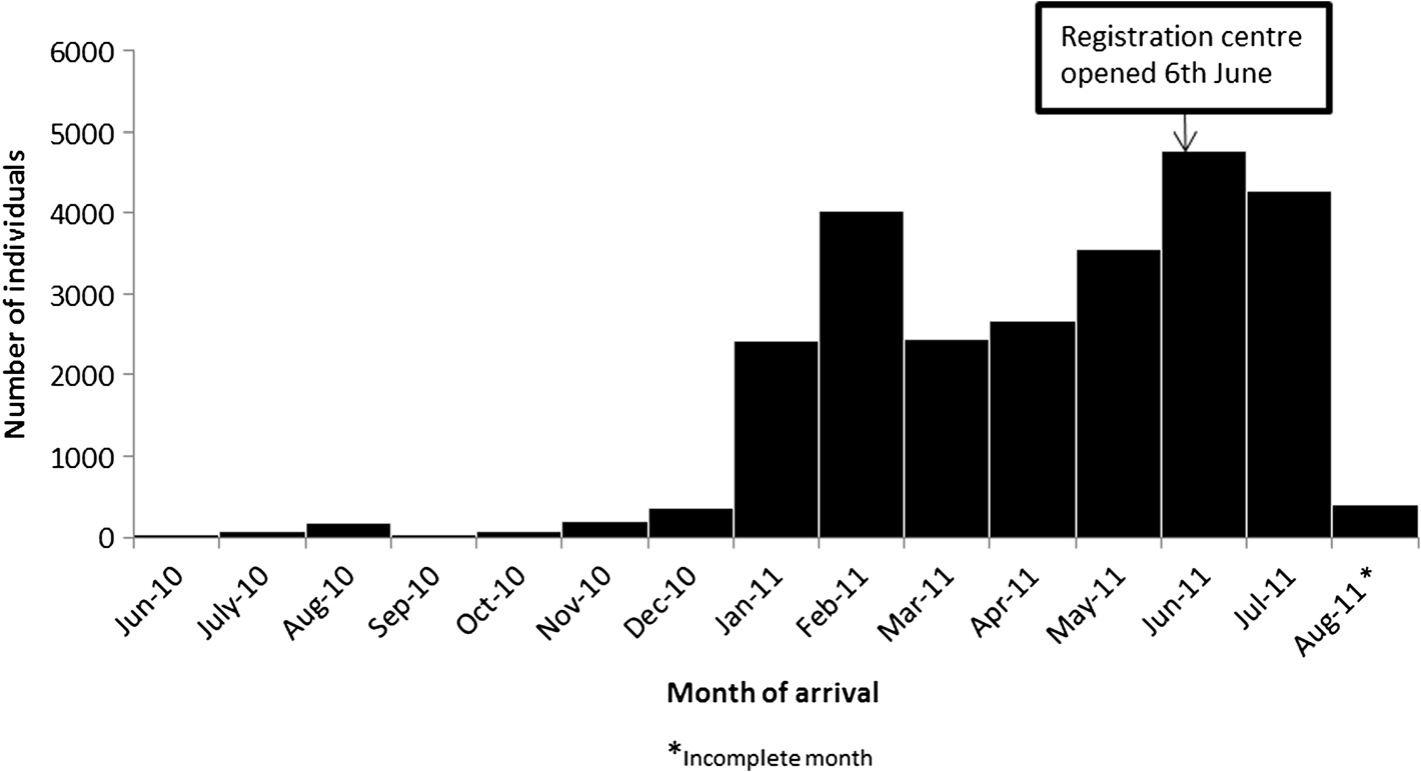
Number of individuals arriving, by month, at Bulo Bacte, Dagahaley refugee camp, Dadaab, Kenya, 01 June 2010 – 10 August 2011 [N = 25,503].

Figure 1 shows the month of arrival of individuals in BB.

There were 360 deaths reported during the 177 days of the recall period, of which 186 (52%) were among children aged under 5 years (Table 1). Of these deaths, 25 (7%) were among children born during the recall period. The median age of the deceased was 4 years (Interquartile range: 1–20).

**Table 1:** Number of deaths (%) by age and by place of death (N = 360), period 15 February – 10 August 2011, Bulo Bacte, Dagahaley refugee camp, Dadaab, Kenya

**Age (years)**	**Before arriving in Dagahaley (%)**	**In Dagahaley (%)**	**Overall (%)**
**<1**	25 (23.2)	46 (18.3)	**71 (19.7)**
**1-4**	30 (27.8)	84 (33.5)	115* (32.0)
**5-14**	10 (9.3)	62 (24.7)	**72 (20.0)**
**15-29**	9 (8.3)	14 (5.6)	**23 (6.4)**
**30-44**	9 (8.3)	13 (5.2)	**22 (6.1)**
**45-59**	11 (10.2)	8 (3.2)	**19 (5.3)**
**60+**	14 (13.0)	24 (9.6)	**38 (10.6)**
**Total**	**108 (30.0)**	**251 (70.0)**	**360 (100)**

* Information on place of death is missing for 1 individual.

The crude death rate for the entire recall period was 0.8 per 10,000 person-days; this disaggregated to 0.6 and 1.0 per 10,000 person-days for the periods before and after arriving in BB, respectively. The under-5 death rate was 1.8 per 10,000 person-days; this disaggregated to 1.3 and 2.2 per 10,000 person-days for the periods before and after arriving in BB, respectively.

**Figure 2:**
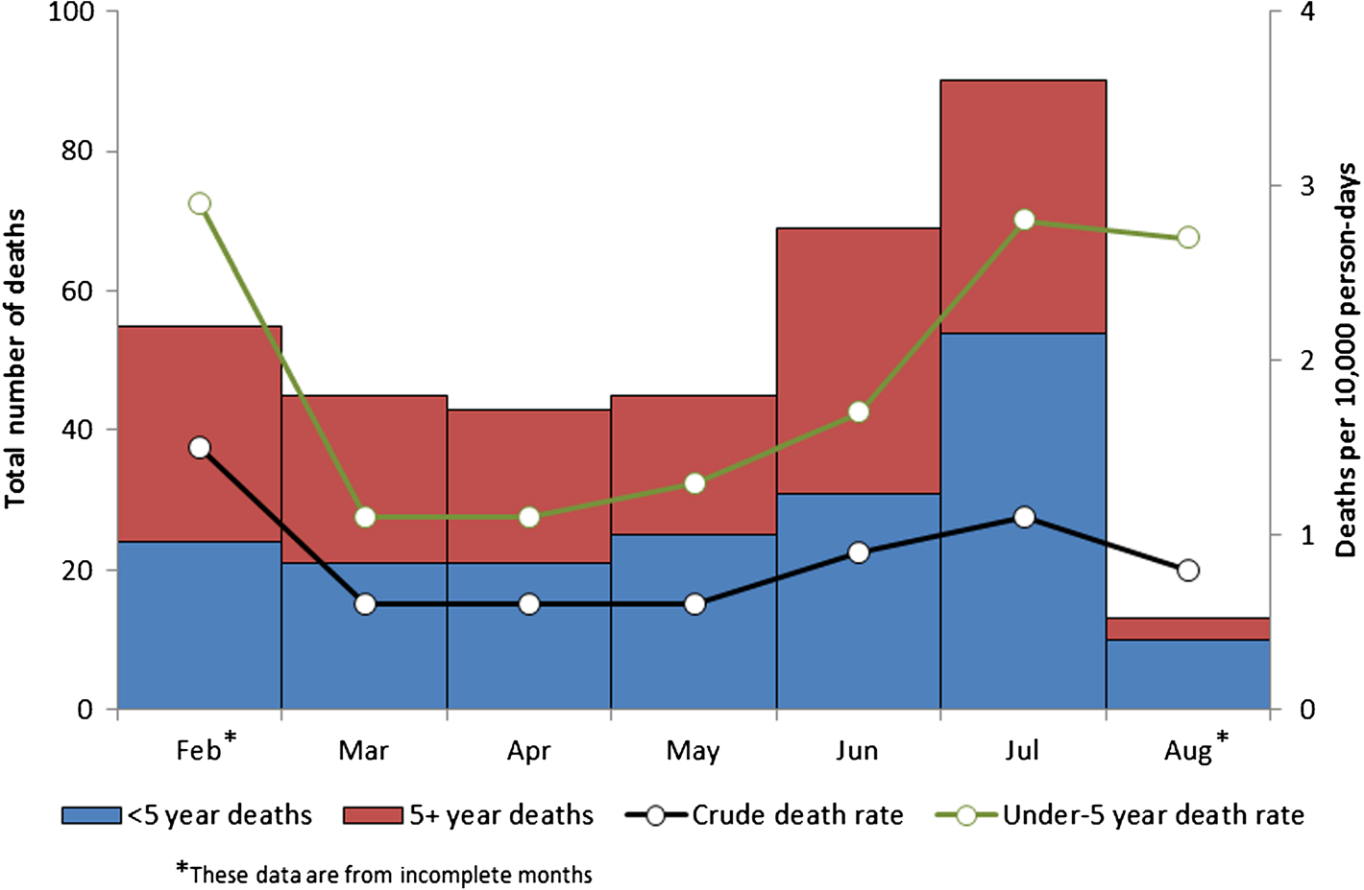
Total and under-5 monthly deaths (left axis) and associated crude (CDR) and under-5 death rates (U5DR) per 10,000 person-days (right axis), period 15 February – 10 August 2011, Bulo Bacte, Dagahaley refugee camp, Dadaab, Kenya.

The disaggregation of deaths according to calendar month is shown in Figure 2. The death rates show a ‘U-shaped’ curve, being elevated above the emergency thresholds in February, dropping below-emergency levels between March and May, and rising to above-emergency levels again by July. The disaggregation of deaths and death rates according to time since arrival in BB (three groups: less than 3 months; between 3 and 6 months; and more than 6 months) is shown in Table 2. A trend of decreasing crude and under-5 death rate with length of stay in BB was observed, from 1.5 and 2.5 deaths per 10,000 person-days (CDR and U5MR, respectively) among the most recent arrivals in BB, to 0.6 and 1.4 deaths per 10,000 person-days (CDR and U5MR, respectively) among the longest-established refugees in BB.

**Table 2:** Number of deaths, and crude and under-5 death rates, by duration of stay in camp, period 15 February – 10 August 2011, Bulo Bacte, Dagahaley refugee camp, Dadaab, Kenya

**Mortality measure**	**Duration of stay in camp at survey date**			Overall*
	**<3 months**	**3-6 months**	**>6 months**	
**Crude death rate per 10,000 person-days (deaths/person-days)**	1.5	0.9	0.6	**1.0**
	(90/622429)	(123/1302210)	(31/562203)	**(251/2486842)**
**Under 5 death rate per 10,000 person-days (deaths/person-days)**	2.5	2.2	1.4	**2.2**
	(36/143384)	(70/314547)	(20/146681)	**(130/604612)**

* information on duration of stay is missing for 7 individuals, 4 of whom are children aged under 5 years.

**Figure 3:**
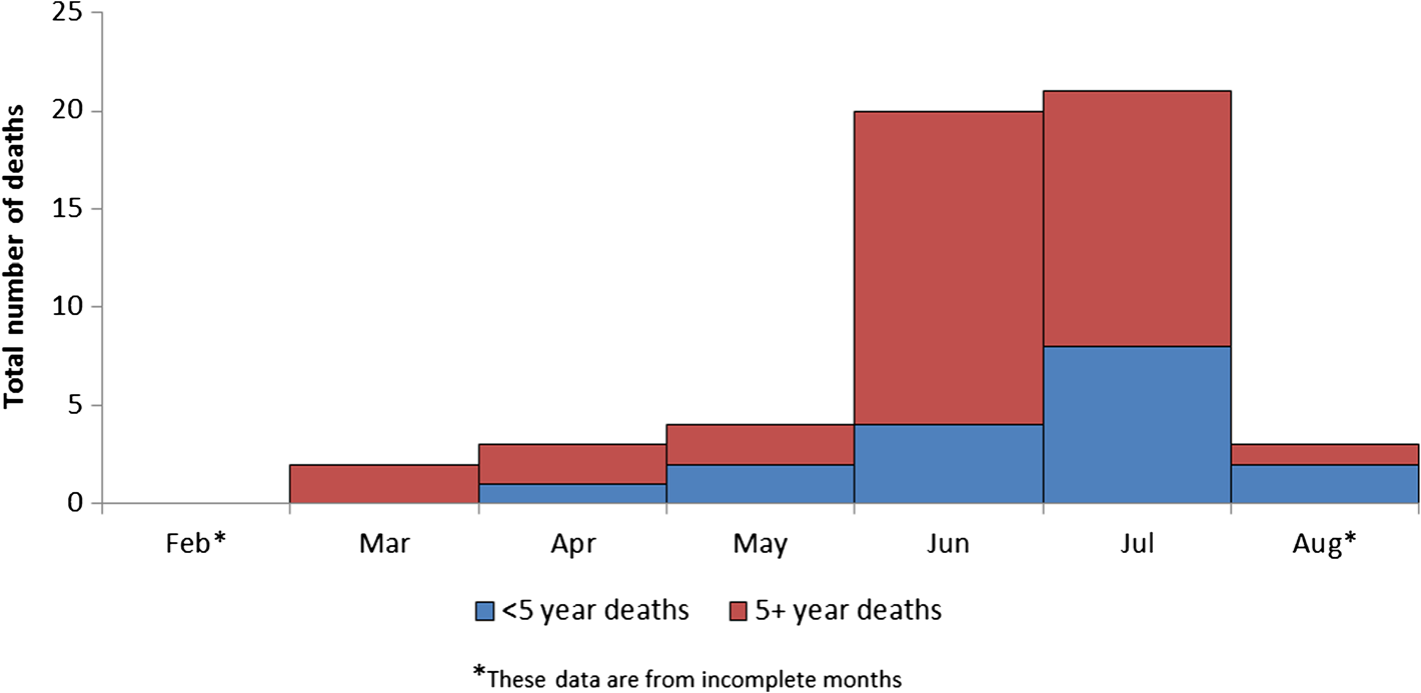
Total and under-5 monthly deaths attributed to measles after arrival at Bulo Bacte, period 15 February – 10 August 2011, Dagahaley refugee camp, Dadaab, Kenya.

More than two-thirds of all deaths were reported to have been associated with diarrhoea (25%), cough or other breathing difficulties (24%), or with fever (19%) (Table 3). Measles accounted for a reported 17% of all deaths; this was due to a measles outbreak that occurred between June and October 2011. Figure 3 shows the deaths attributed to measles, according to month. If deaths attributed to measles are excluded, the crude and under-5 death rates after arrival at BB were 0.8 and 1.8 deaths per 10,000 person-days, respectively.

**Table 3:** Reported causes of death as percentages of total deaths (overall and according to place of death), period 15 February – 10 August 2011, Bulo Bacte, Dagahaley refugee camp, Dadaab, Kenya

**Cause of death**	**Before arriving in Dagahaley (%)**	**In Dagahaley (%)**	**Overall (%)**
**Diarrhoea**	21 (19.8)	66 (26.8)	**87 (24.6)**
**Cough/breathing difficulties**	27 (25.5)	56 (22.8)	**83 (23.5)**
**Fever with/without shivering**	30 (28.3)	38 (15.5)	**68 (19.3)**
**Measles**	4 (3.8)	55 (22.4)	60* (17.0)
**During or just after childbirth (within 1 month)**	6 (5.7)	4 (1.6)	**10 (2.8)**
**Malnutrition**	1 (0.9)	8 (3.3)	**9 (2.5)**
**Accidental trauma**	4 (3.8)	1 (0.4)	**5 (1.4)**
**During pregnancy**	1 (0.9)	3 (1.2)	**4 (1.1)**
**Intentional violence**	2 (1.9)	2 (0.8)	**4 (1.1)**
**Other**	2 (1.9)	3 (1.2)	**5 (1.4)**
**Unknown**	8 (7.5)	10 (4.1)	**18 (5.1)**
**Total**	**106 (100)**	**246 (100)**	353** (100)

* Information on place of death is missing for 1 individual.

** Information on cause of death was missing for 7 individuals.

GAM was observed in 13.4% (956/7144), and SAM in 3.0% (212/7144), of children measuring 67-< 110 cm (Table 4). Among children of height 67-< 87 cm, these figures were 23.6% (GAM) and 6.0% (SAM); among children of height 87-< 110 cm, these figures were 6.4% (GAM) and 0.9% (SAM). Among children measuring 110-< 140 cm, 9.8% (353/3597) met the admission criteria for entry into the nutritional programme. 62 (0.6%) children presented with bipedal oedema: 58 (0.8%) among those of height 67-< 110 cm and 4 (0.1%) among those of height 110-< 140 cm.

**Table 4:** Prevalence of acute malnutrition among children measuring 67-< 140 cm, by duration of stay in camp, Bulo Bacte, Dagahaley refugee camp, Dadaab, Kenya, August 2011

**Height**	**Degree of malnutrition**	**Duration of stay in camp at survey date**			**Cochran-Armitage test-for-trend**	**Overall%**
		**<3 months**	**3-6 months**	**>6 months**		**(n/N)**
		**% (n/N)**	**% (n/N)**	**% (n/N)**		
**67 - <110 cm**	**Severe acute malnutrition**	3.2	3.9	2.0	1.19	**3.0**
		(110/3441)	(71/2359)	(19/949)	p = 0.09	**(212/7144)**
	**Global acute malnutrition**	15.4	11.9	9.4	1.31	**13.4**
		(529/3441)	(279/2359)	(89/949)	p < 0.001	**(956/7144)**
**67 - <87 cm**	**Severe acute malnutrition**	6.6	5.9	4.0	1.23	**6.0**
		(93/1403)	(55/935)	(16/402)	p = 0.06	**(173/2893)**
	**Global acute malnutrition**	25.6	22.2	18.1	1.23	**23.6**
		(359/1403)	(207/935)	(73/402)	p = 0.001	**(682/2893)**
**87 - <110 cm**	**Severe acute malnutrition**	0.8	1.1	0.5	1.03	**0.9**
		(17/2038)	(16/1424)	(3/547)	p = 0.89	**(39/4251)**
	**Global acute malnutrition**	8.3	5.1	2.9	1.61	**6.4**
		(170/2038)	(72/1424)	(16/547)	p < 0.001	**(274/4251)**
**110- < 140 cm**		11.0	9.2	6.2	1.29	**9.8**
		(193/1749)	(109/1189)	(28/454)	p = 0.002	**(353/3597)**

A trend of decreasing malnutrition prevalence with length of stay in BB was observed (Table 4). Among children of height 67-< 87 cm, this trend was strongly significant for GAM (p = 0.001), and showed a tendency for SAM (p = 0.06). Among children of height 87-< 110 cm, this trend was strongly significant for GAM (p < 0.001), but there was no relationship for SAM (p = 0.89). Among children of height 110-< 140 cm, there was a strongly significant trend of decreasing proportion of children meeting the admission criteria for entry into the nutritional programme with length of stay in BB (p = 0.002).

Of the 619 cases of suspected measles detected in Dagahaley, 256 (41%) originated in BB, of which half (49%) were male. The median age of suspected cases originating in BB was 23 years [Interquartile range: 15 – 30 years; range: 1 – 56 years].

## Discussion

This survey revealed an alarming situation; both crude and under-5 death rates, and the prevalence of SAM and GAM, were at emergency levels in the outskirts of a refugee camp which was served by several international NGOs and UN agencies [8].

The recall period coincided with an outbreak of measles in Dagahaley, which began in June and continued until October 2011 [9], and which partially explains the increasing under-5 death rate observed; 17% of all deaths were reported to have been caused by measles. As a result of the two decades-long civil war in Somalia, vaccination coverage among all ages has declined to the point that outbreaks of infectious diseases such as measles are increasingly likely. Indeed, there was a simultaneous measles outbreak among newly-displaced Somalis in Kobe refugee camp in Ethiopia and in several places in Somalia, including the capital Mogadishu [10,11]. Outbreaks of measles among populations in crisis are common and well-documented, such that the routine vaccination of children aged 6 months to 15 years, supplemented by mass vaccination campaigns, is widely accepted as one of the most important public health interventions for averting preventable morbidity and mortality among crisis-affected populations [8,12-14].

**Figure 4:**
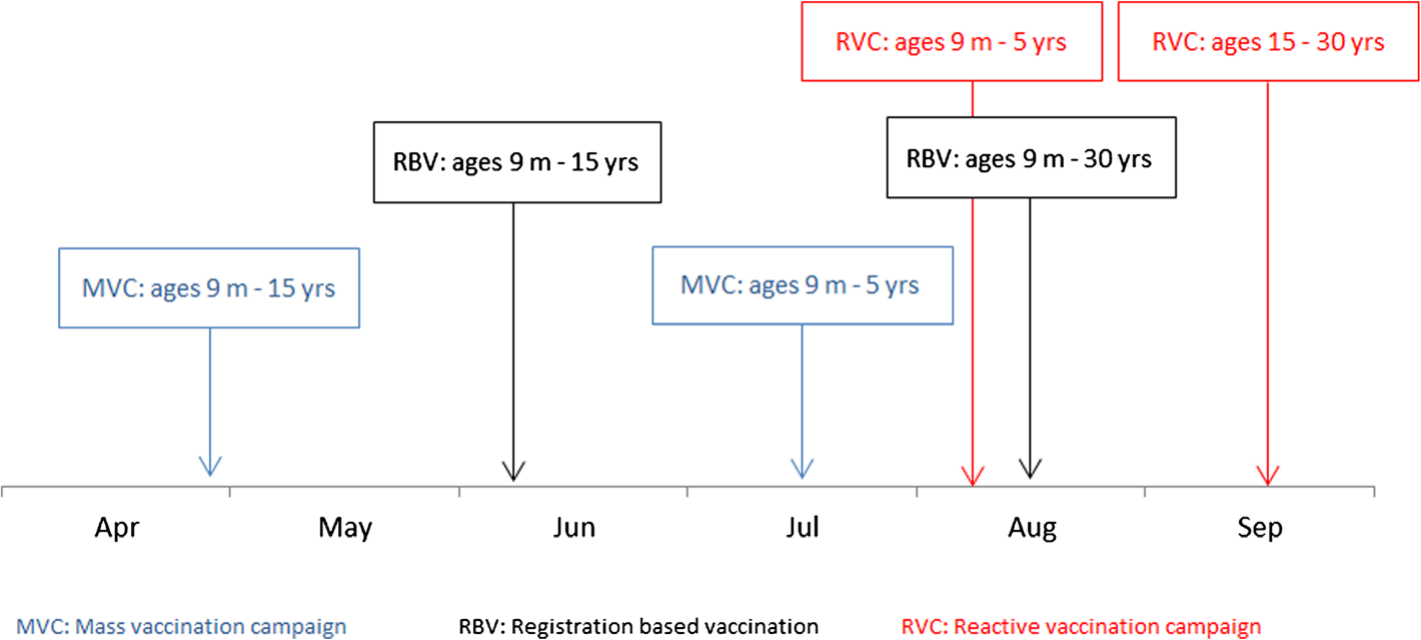
Timeline of measles vaccination interventions conducted between April and September 2011, Bulo Bacte, Dagahaley refugee camp, Dadaab, Kenya.

Figure 4 shows a timeline of various measles vaccination interventions conducted in BB. A mass measles vaccination campaign was organised at the end of April 2011, targeting children aged between 9 months and 15 years, with a follow-up campaign in July 2011 to vaccinate those children aged under 5 who did not receive measles vaccination in April. When a registration centre was opened within Dagahaley in June 2011, all children aged 9 months to 15 years were routinely vaccinated against measles upon registration. In response to the outbreak, a reactive vaccination campaign (RVC) targeting children under 5 years was launched throughout Dagahaley camp in early August, and the target age group for vaccination at the registration centre was increased to 30 years. In September 2011, a RVC was organized targeting individuals aged 15 to 30 years. It is worth noting that the RVC launched in August 2011 ran concurrently with the survey described in the article, and therefore the decision was taken not to include measles vaccination coverage in the survey, both because the results would have had no influence over any decision to launch such a campaign, and because the coverage at the end of the survey would have been different to the coverage at the start, thereby rendering the results immediately invalid. UNHCR and partner organisations assessed measles vaccination coverage in BB shortly after this campaign, and reported a coverage of 83.9% (95% CI: 73.7 – 94.0%) among children aged 9–59 months [15]. 

This measles outbreak was preventable; the essential lessons from past mass displacements should have been learned, and a suitable aggressive vaccination strategy implemented at an earlier stage [16-19]. We report that, if the deaths reportedly due to measles are excluded from the calculation, death rates after arrival at BB fall from 1.0 to 0.8 per 10,000 person-days (CDR), and from 2.1 to 1.8 per 10,000 person-days (U5DR). In other words, it could be argued that it was these deaths, attributed to the measles outbreak, which elevated the death rates to above the emergency threshold. However, this supposition assumes that those individuals who died from measles otherwise had zero risk of death; as severity of measles is influenced by nutritional status, we believe that this assumption is not valid and therefore that this would be an incorrect and unfair conclusion [20,21].

In mitigation, the population most affected was that which had recently arrived, containing a large proportion of families unregistered by camp management due to the overwhelming arrival rate of these refugees. The late establishment of the registration centre and vaccination at arrival permitted the development of a pool of susceptible individuals in BB. In addition, the measles outbreak in Dagahaley was characterized by an unusual age distribution; the median age of patients recorded in the outbreak line list was 23 years, with 75% of patients aged 15 years or older, suggesting that a wider age group could have benefitted from vaccination, an observation reported earlier following a measles outbreak in a refugee camp in Tanzania [16]. However, the current ‘one-size-fits-all’ recommendations are to vaccinate all children aged 6 months to 15 years, and do not take into account the context-specific epidemiology, which in this case included a highly immunologically-naïve population due to the breakdown of health-care services arising from the ongoing political crisis in Somalia [22,23].

Early identification of the unusual age-distribution of measles cases would have helped guide vaccination policy in this setting. Indeed, the disaggregation of deaths attributed to measles by age and by month (Figure 3) shows that age distribution of measles cases was detectable in June 2011, at an early stage of the epidemic. However, this would have required information that was not available at the time: low health facility utilisation rates and under-resourced community-based surveillance of epidemic-prone diseases meant that most measles cases occurring before July were not detected. In July 2011, by which time an outbreak had been declared and active community-based surveillance strengthened, more data were available which led MSF to advocate for a wider target age group for the RVC planned for early August, but this advocacy was unsuccessful due to the limited resources available for that particular campaign. The target age group for vaccination at registration was, however, expanded to include all individuals aged 9 months to 30 years. Owing to the failure of the August RVC to halt the epidemic, which peaked in August and September [9], adults aged 15–30 years were the target age group for the subsequent RVC.

More recent arrivals were in a significantly worse state, which was reflected both in death rates and in nutritional status. We found trends of decreasing death rates with length of stay in BB, such that those residents who had arrived more than six months prior to the survey date had death rates well below the emergency thresholds, while those who had arrived within three months of the survey date had death rates which were above the emergency thresholds. Individuals who had been resident in BB for an intermediate length of time were found to have death rates at an intermediate level.

Similarly, we found a higher prevalence of acute malnutrition and children meeting the admission criteria for entry into the nutritional programme among those children who arrived during the three months prior to the survey than among those children who arrived earlier. The same pattern was reported in a subsequent survey conducted in BB [15].

This apparent improvement in health and nutritional status over time may be due to the assistance gained after registration and the development of coping strategies, but may also be due in part to:

a) a high concentration of deaths in the period immediately prior to the survey date (in particular, due to the measles outbreak), which may have resulted in artificially elevated death rates among recent arrivals due to the relatively low number of person-days contributed by these individuals (in other words, a low denominator rather than a high numerator used in the calculation of death rates); and

b) higher mortality among those children with poor nutritional status on arrival: both recovery and death have the effect of decreasing the prevalence of malnutrition by removing these children from the numerator in the calculation of malnutrition prevalence. Those malnourished children who had recently arrived had had less time in which to reach either of these outcomes.

Delays in registration and food distribution were reported by many residents, and may partially account for the high mortality observed among residents of BB. We report higher death rates among individuals after having arrived in BB than before; although this suggests that conditions are worse for individuals once they have arrived, the death rates prior to arrival are subject to selection bias. Therefore, while the death rates reported for the period within the camp should be considered to be reflective of the experience of the population while in the camp, those rates reported for before arrival should not be considered generalizable to the population of Somalia during the recall period.

One limitation of this study is that we did not use a validated verbal autopsy technique when obtaining information on cause of death as this was not a principal objective, and therefore these results should be interpreted with caution. We did not use standard case definition for measles deaths (any death within one month after rash onset); it is likely that some of the deaths associated with diarrhoea, cough or breathing difficulties and fever were in fact cases of measles. Event calendars and height sticks were used to approximate events and ages, which can lead to misclassification. However, less recall bias is expected for the most important events, such as deaths of household members [5].

Another survey conducted in September 2011 reported higher levels of malnutrition and mortality in BB than we observed [15]. This may be due to the measles epidemic, which reached its peak in August and September; it is frequently reported that levels of malnutrition are increased in the weeks following an outbreak [20].

## Conclusion

This survey revealed unacceptably high death rates and prevalence of malnutrition in the outskirts of a long established refugee camp, albeit among a recently-arrived population. Although the levels of malnutrition may be partly explained by the poor health of new arrivals, the high mortality among refugees after their arrival in Dagahaley reflects a failure of the various humanitarian and governmental actors to adequately safeguard the welfare of this population. While the massive influx of refugees did pose enormous difficulties, outbreaks of measles and long delays before registration (which permits access to food distributions) should not have occurred. The recommendations for measles vaccination among crisis-affected populations should be revised to take into account the epidemiologic context [24].

These results highlight the necessity to rapidly detect the acute worsening of a protracted crisis, combined with the prompt adjustment and scaling-up of programmes (from routine activities to incorporating emergency response) at the earliest signs of such a worsening. Organisations charged with the responsibility of providing services for vulnerable people must be sensitive and reactive to changes among the population they assist.

## Competing interests

The authors declare that they have no competing interests.

## Authors’ contributions

All authors contributed to the conception of the study. JP, AR and KP designed and conducted the study. All authors contributed to the interpretation of the results. JP drafted the manuscript, and all other authors critically revised the manuscript for submission. All authors read and approved the final manuscript.
